# Impacts of non-native invertebrates and plants on polar soil systems

**DOI:** 10.1038/s44185-026-00127-8

**Published:** 2026-05-22

**Authors:** Octavia D. M. Brayley, Peter Convey, Sami Ullah, Scott A. L. Hayward

**Affiliations:** 1 School of Biosciences, University of Birmingham, Birmingham, UK; 2 British Antarctic Survey, NERC, High Cross, Cambridge, UK; 3 Department of Zoology, University of Johannesburg, Auckland Park, Johannesburg, South Africa; 4 Biodiversity of Antarctic and Sub-Antarctic Ecosystems (BASE), Santiago, Chile; 5 The Birmingham Institute of Forest Research, University of Birmingham, Edgbaston, Birmingham, UK; 6 School of Geography, Earth and Environmental Sciences, University of Birmingham, Edgbaston, Birmingham, UK

**Keywords:** Ecology, Ecology, Environmental sciences, Microbiology

## Abstract

Invasive non-native species are amongst the most serious threats to biodiversity at local and global scales. Due to their geographical remoteness, extreme conditions and lower levels of human activity, the Earth’s polar regions have seen fewer invasions to date compared to temperate and tropical areas. However, increasing human activity in high latitude areas brings the risk of many more species introductions, while climate warming is reducing many of the abiotic barriers to species establishment. Polar ecosystems are particularly susceptible to the negative effects associated with invasive species due to their low native diversity, simple food chains and the availability of apparently vacant niches. To date, few studies have tested the effects of non-native species on soil properties in the typically nutrient-limited polar regions. Non-native arthropods and plants may introduce their novel microbiomes and fungal endophytes to a new environment, and this can lead to changes in organic matter decomposition and levels of bioavailable nutrients such as nitrogen. Decomposition rates may be increased further in synergy with climate warming, releasing locked up nutrients in addition to nutrient enrichment facilitated by invasive species. Enhanced nutrient availability and microbial activity may, in turn, create more favourable conditions for the establishment and, for some, the subsequent invasion of further non-native species, as well as potentially benefiting native arthropod and plant communities. This review characterises the interactions between invasive species and global change, highlighting current and predicted future impacts on soil health in polar ecosystems. In addition, we identify priority areas for further research to better understand these impacts and guide management practices.

## Introduction

## Non-native plants and invertebrates in the polar regions

**Fig. 1:**
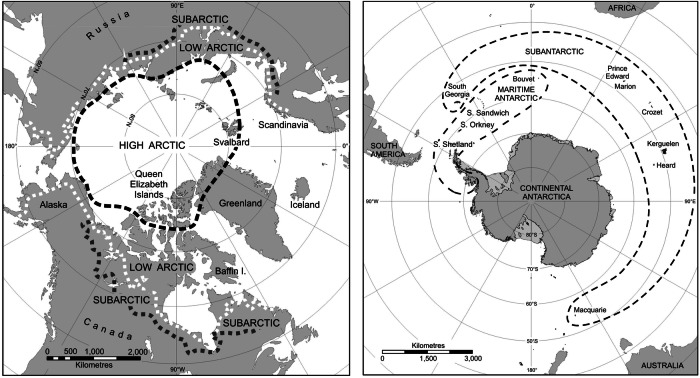
Map showing Arctic biogeographic zones (left): delineation of the High Arctic, Low Arctic, and Subarctic regions across the Northern Hemisphere. The High Arctic zone is indicated by the bold dashed line. Figure produced by the Mapping and Geographic Information Centre, British Antarctic Survey. Map illustrating the major biogeographic zones of Antarctica (right): Maritime Antarctic and Continental Antarctic, as well the surrounding Sub-Antarctic Southern Ocean islands. Figure from Convey 2010^158^, with permission.

The arrival of organisms beyond their natural range is generally directly or indirectly associated with human activity^1,2^. A proportion of non-native species can become ‘invasive’, meaning they integrate into the native ecosystem expand their range, resulting in negative impacts on native species (e.g., by outcompeting) and, potentially threatening human, animal, plant or ecosystem health^3^. Non-native species are now considered one of the greatest threats to global biodiversity^4,5^, including in the Earth’s polar regions^6–8^. Globally, introductions of non-native species have predominantly occurred accidentally, for instance in association with tourism^9–11^, trade^12–14^, and through ballast water in marine environments^15–18^. Isolated High Arctic regions and Antarctica (Fig. 1) have seen fewer non-native introductions to date compared to other global regions^6–8,19,20^, a result of their geographical remoteness, extreme climates and relatively low levels of human activity^21,22^.

**Fig. 2:**
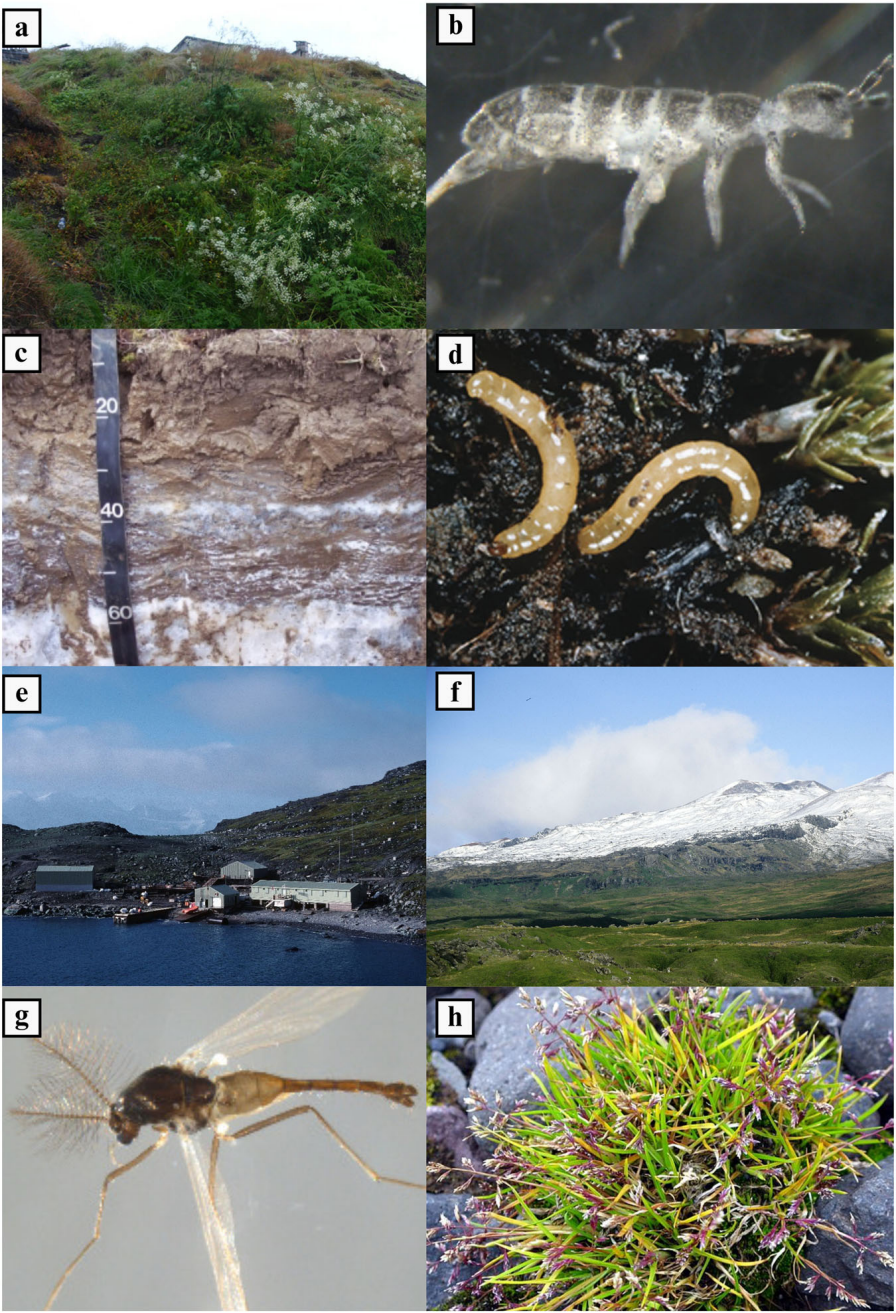
Examples of non-native species and their vegetation/soil habitats in the Arctic and Antarctic. **a** Vegetation growing on the imported nutrient-rich soils of Barentsburg in Svalbard (image obtained from Coulson et al. (2013)^27^, Creative Commons CC BY-NC 4.0). **b** *Desoria grisea*, a non-native collembolan present in imported soils at the settlement of Barentsburg, Svalbard (image: David Porco, Creative Commons 2010). **c** Soil from Ellef Ringes Island, Nunavut, Arctic Canada that has been impacted by permafrost melting, potentially increasing rates of microbial respiration and releasing carbon dioxide into the atmosphere (image obtained from Ping et al. (2015)^159^, Creative Commons CC BY-NC 4.0). **d** The detritivorous larvae of *Eretmoptera murphyi*, a non-native chironomid midge present on Signy Island, maritime Antarctic (image: British Antarctic Survey). **e** View of Signy Island and the British Antarctic Survey research station, close to where *E. murphyi* was first introduced to the island (arrow) (image: British Antarctic Survey). **f** Terrestrial habitats of sub-Antarctic Marion Island (image from Smith & Mucina (2006)^160^, with permission). **g** *Limnophyes minimus*, a non-native chironomid midge present on sub-Antarctic Marion Island (image: NTNU Museum of Natural History and Archaeology, Creative Commons 2012). **h** *Poa annua*, a non-native grass species established in the sub-Antarctic and part of the maritime Antarctic (image from Znoj et al. (2017)^161^, with permission).

In Antarctica, scientific research, its support operations and tourism have been the largest contributors to non-native introductions^8,23^. For instance, between 2007 and 2008, Chown et al. (2012) reported that 31,732 non-native seeds had been unintentionally transported by tourists and scientific operational staff to Antarctica on their clothing, and 38,897 seeds on equipment^24^. This is a particularly important issue given that terrestrial habitats are restricted to very few coastal areas of Antarctica and many of these sites are primary locations for tourism and scientific research. In the entire Antarctic continent, only 18 non-native species are currently formally reported to have established, all within the maritime Antarctic, with just one species of terrestrial invertebrate and one plant currently considered invasive^8^. However, the number increases to >200 if the much milder sub-Antarctic islands are included^6,7,25^ (Fig. 1). In High Arctic Svalbard (Fig. 1), there are currently known to be 98 non-native plant species established^20^. Ware et al. (2012) recorded more than 1000 seeds, representing 53 species, on the footwear of 259 travellers to Svalbard over one summer, with 26% of the seeds germinating under local conditions^26^. Again, on Svalbard, grass seeds and nutrient-rich soil imported from Ukraine (in the then Soviet Union) was transferred to Barentsburg for agriculture and to create green spaces (Fig. 2a). This is thought to have led to the introduction of five species of non-native annelid worm (*Cognettia glandulosa, Enchytraeus dichaetus, Dendrodrilus rubidus, Dendrobaena hortensis* and the recently discovered *E. buchholzi*), two species of non-native mite (*Paragamasus insertus* and *Vulgarogamasus remberti*) and five species of non-native Collembola (*Hypogastrura purpurescens, H. assimilis, Deuteraphorura variabilis, Folsomia fimetaria* and *Desoria grisea*)^27,28^ (Fig. 2b). While all these species are now established in these unusually nutrient-enriched areas, they do not appear to have spread beyond them. However, it has been predicted that some Collembola may be capable of colonising other areas of native soils and vegetation that also have nutrient-rich ornithogenic soil, such as beneath bird breeding cliffs or along seashores^29^.

The larger number of non-native species in the High Arctic relative to the maritime and continental Antarctic is most likely due to much greater human activity in the northern region (particularly in the last 100 years). This increases both propagule pressure and habitat disturbance which, combined with low competition from native species and amplified rates of climate warming, provides pathways for further non-native establishment^30^. Indeed, the Arctic region has been exposed to regular natural and human-mediated colonisation pathways since the end of the last glacial maximum, much more so than the more isolated Antarctic. This longer timescale makes it difficult to disentangle the contributions of natural vs. anthropogenic dispersal in these northern regions. Furthermore, our understanding of native Arctic invertebrate communities remains limited^31^, making it challenging to determine whether a species is truly non-native or merely uncommon.

Moving beyond knowledge of non-native invertebrates and plants, while there is reasonable understanding of microbial taxonomic and functional diversity in Arctic^32^ and Antarctic^33^ soils, little is currently known about the microbiomes associated with native plants and invertebrates. Furthermore, no studies to date have considered how the microbiomes of non-native invasive species might impact biogeochemical cycling or other processes in polar soil systems. Polar terrestrial ecosystems have the potential to be particularly susceptible to the negative effects associated with invasive species due to their low native diversity. ‘Simple’ food chains typically comprise only a few trophic levels (often dominated by microbes) and may often have vacant niches, for instance with the absence of larger terrestrial invertebrate herbivores or predators^25,34^. Polar terrestrial species also commonly show well-developed features of adversity selection, with multiple stress tolerance adaptations^35^, which comes at the cost of reduced competitive ability, potentially accentuating vulnerability to incoming more competitive species.

This review highlights the critical importance of investigating species microbiomes in order to understand both the capacity of non-native species to become established and their broader impacts on polar soil systems. We integrate the predicted effects of climate change, in particular on organic matter decomposition rates and soil respiration, as well as emphasizing the need for more biologically-relevant microclimatic data. We then discuss a series of case studies to place in context current understanding of bottom-up effects of non-native species on polar terrestrial communities, soil biochemistry, nutrient cycling and primary productivity. These topics are further considered in the context of terrestrial biodiversity and habitat conservation in polar regions.

## Non-native microbiomes

Whenever any non-native plant or invertebrate is introduced to a new location, its associated microbiome is transported concurrently. The microbiomes of plants and arthropods can be very complex^36,37^ and alter the structure of the native soil microbial communities^38^. Microbiomes are known to be important for host survival under stressful conditions^39,40^, with a clear role in allowing non-native species to successfully establish and adapt to new environments^41,42^. Non-native species often have taxonomically and functionally more diverse microbiomes than their native counterparts, which may also provide a competitive advantage^43^. Additionally, parts of the microbiome can influence nutrient cycling in the soil^38,42^, both directly through their own metabolic activity in decomposition and nutrient transformation, and indirectly by enhancing host fitness and altering litter inputs that affect soil chemistry^44^. The legacy effects of non-native species on nitrogen cycling, in particular, cannot be disentangled from their associated microbiomes, because nitrification is a microbial-mediated process that drives the conversion of ammonium (NH_4_^+^) into nitrate (NO_3_^−^).

Candidate bacterial and fungal species that may enhance nutrient cycling in the Arctic and Antarctic (i.e. species that have previously been reported in polar soils and have known nutrient cycling properties) include members of the phyla Cyanobacteria, Actinobacteria, Bacteroidetes, Proteobacteria, Chloroflexi, Basidiomycota and Ascomycota^45–49^. Some Archaeal phyla may also contribute to nutrient cycling, such as the Crenarchaeota^48^. Many of these taxa have also been reported in invertebrate gut or whole body microbiomes in Antarctica^50–53^. However, currently, no studies have linked the introduction of non-native arthropods or plants and their associated microbiomes to alterations in soil biogeochemistry in either polar region. This is an area that critically requires further research, given there is a unique polar microbial diversity that is currently under-represented in the literature^54^. Further research is also required to develop in-depth understanding of invasion mechanisms and the health of polar terrestrial ecosystems, as well as underpinning the development of stricter biosecurity protocols that take microbial communities into consideration.

## Climate change and climate data

Abiotic stresses, particularly low temperature and limited moisture availability, are important barriers to the establishment of non-native species transported to polar environments. However, parts of the polar regions are warming considerably more rapidly than the rest of the globe^55–59^. This ‘polar amplification’ is partly driven by diminishing sea ice and land ice/snow cover, resulting in less reflection and higher absorption of solar energy by the ocean and land surface, leading to higher temperatures and therefore a higher rate of ice and permafrost melt, termed the “surface albedo feedback”^60^. The melting of permafrost not only increases water availability, but also releases carbon dioxide and methane into the atmosphere, further compounding increases in atmospheric temperature which, in turn, increases the abundance of microalgae, microbial activity and the rate of nutrient cycling in wet environments^61,62^. The combined consequences alter local ecosystems, modifying conditions and facilitating the establishment of non-native species^63–67^.

The amplifying effects of global warming are strongest in the Arctic, with recent research showing that parts of this region have warmed four times more than anywhere else on Earth over the last 43 years^68^. In addition, satellite images available since the late 1970s indicate that September Arctic sea ice extent has been reducing by about 12.8% per decade over the last 41 years, compared to the 1981–2010 average^69^. In the Antarctic, parts of the Antarctic Peninsula have shown some of the greatest warming trends, for example increases of 0.46 °C ± 0.15 °C per decade between 1951–2018 at Faraday/Vernadsky Station^70^, and the highest air temperature recorded south of 60°S has also been recorded in the Maritime Antarctic, 19.8 °C in 1982 on Signy Island^71^. Warming is generally thought to have a positive influence on non-native species in polar regions, for example increasing seed (propagule) germination^72^ and providing more degree days (cumulative energy receipt) for plant growth^73^. Similar advantages are predicted for non-native invertebrates that are transported to polar regions. For example, while many polar species have evolved multi-year life cycles as an adaptation to very short summer seasons^35,74^, longer and warmer growing seasons provide an opportunity for univoltine (or even some multi-voltine) non-native species to establish and increase their populations at a faster rate.

To fully understand the potential impacts of climate warming on polar soil systems, microclimate data at scales relevant to soil biogeochemical processes and resident organisms are required^73^. This is crucial for predicting the invasive potential of non-native species, as well as calculating the rates of biochemical processes and growing degree days (for phenology and population growth models). Unfortunately, in situ soil temperature monitoring is very limited in terms of spatial and temporal coverage in both the Arctic^75^ and Antarctic^76^ and these regions were largely missing from the global analysis of Lembrechts et al. (2021), which identified an annual mean soil temperature offset of +3.6 ( ± 2.3°C) in cold and dry biomes globally^77^. However, this study remains important in highlighting the degree of difference in soil conditions relative to broader climate data. Chaves et al. (2017) recorded soil temperatures across different sites on King George Island (maritime Antarctic) at a range of depths^76^. Their data indicated an air-soil temperature offset of >2.0 °C, as well as a significant lag between changes in air and soil temperature. In addition, the authors reported different patterns of change across seasons (summer cooling and winter warming) and considerable spatial variability in freezing and freeze-thaw events. Convey et al. (2018) similarly showed significant differences between air temperature and temperatures in a range of terrestrial microhabitats in both the Arctic and Antarctic^73^.

The availability of soil moisture data is even more limited than that of soil temperature data in the Antarctic or High Arctic. While satellite microwave soil moisture retrievals from the Soil Moisture Active Passive mission are considered reliable^78^, their course spatial resolution means that they miss important complexity in high latitude systems, including the tight link between water drainage, the presence and depth of permafrost, as well as type and extent of vegetation cover^79^. Even at the scale of single vegetation habitat patches there can be considerable differences in how climate warming might impact moisture availability^80^. Thus, much more long-term terrestrial microclimate data are required given that abiotic factors, such as temperature and water availability, will be the dominant drivers in determining whether non-native species can establish, the extent of available habitat and how species’ distributions might change in both the Antarctic^80–82^ and Arctic^83^. Both of these factors can also influence nutrient availability, due to their impact on microbial activity, thereby reducing one of the key biotic barriers to non-native species establishment and invasive potential.

## Nutrient limitation and soil biogeochemistry in polar soils

A key knowledge gap in understanding of the impact of non-native species in polar regions is how they can influence soil health^65^. Polar soil systems are typically characterised by limited net primary productivity and macroscopic biodiversity, and an almost complete dominance of ecosystem processes driven by soil microalgae, fungi and prokaryotes to an extent that is rarely seen elsewhere^48,84–87^. Despite this, Antarctic microbial-dominated ecosystems carry out essential carbon capture: 0.5 kg/m^2^ carbon stocks in polar deserts, 3–5 kg/m^2^ carbon stocks in sub-Antarctic tundra and up to 30 kg/m^2^ in penguin nesting sites around the maritime Antarctic^88^. These microbes also undertake nutrient cycling processes, such as nitrogen fixation and mineralization, as well as the maintenance of soil structure and aggregation, thus limiting erosion and run-off^19,65,81,89^. It has been proposed that a 1 °C increase in soil temperature in the Arctic, as well as leading to accelerated permafrost melt (Fig. 2c), will increase microbial respiration rates, resulting in the release of up to 100 megatons of stored carbon per year^90,91^. Although the extent of available ice-free habitats is much less in Antarctica than the Arctic, similar increases in microbial respiration rates and terrestrial decomposition are also expected to take place where these habitats are present^92^. However, as noted in the detailed experimental field manipulation study of Misiak et al. (2021) on southern Alexander Island (southern maritime Antarctic), such increases may be modulated and even reversed depending on the magnitude of warming experienced, with an apparent step change reduction in functional response of the native soil fungal community when soil temperatures reach ~20 °C^93^. Thus, soil surface temperature and associated biochemical changes due to warming may differ between specific locations.

Nutrient cycling in Arctic soils is particularly important for the global climate, as melting permafrost can expose soil containing considerable amounts of soil organic carbon. Permafrost soils contain a resource of up to three times as much carbon in total (1460–1600 billion tonnes) than is currently present in the atmosphere^94^, and microbes can potentially break down this organic carbon source as permafrost retreat takes place, thus releasing carbon dioxide and methane into the atmosphere and providing a positive feedback to the “terrestrial carbon-climate feedback system”^95–97^. Conversely, snow and permafrost melt may increase the abundance and biomass of some moss and lichen species which could then lead to a net uptake of carbon, rather than it being released to the atmosphere^98^. In combination, climate change and future biological invasions will clearly alter soil biodiversity and community structure in polar regions, resulting in important, but currently unknown, changes to soil organic matter decomposition and nutrient cycling processes, the ‘brown food web’ and the overall health of these fragile ecosystems^97,99^.

### Nitrogen limitation

Nitrogen availability is generally limited in polar soils due to the cold climates slowing microbial and nutrient turnover, as well as generally a lack of larger invertebrate detritivores capable of soil mixing and boosting nitrogen mineralisation^23,100,101^. Therefore, plants and microbes in Arctic and Antarctic soils are heavily regulated by the availability of nitrogen via natural processes such as biological nitrogen fixation^48,97^. Increased available mineral nitrogen (nitrate and ammonia) can boost plant and microbial growth, respiration and associated decomposition rates of soil organic carbon, which can release carbon dioxide into the atmosphere when respiration exceeds primary production rates^89^. Conversely, locations in the vicinity of vertebrate aggregations, such as marine vertebrate colonies and resting/moulting areas, and even single nests and bird perching points, can experience higher levels of organic nitrogen because of the hydrolysis of uric acid in the guano and deposition of faeces, feathers/fur, carcasses and food scraps^48,102,103^.

### Phosphorous limitation

Phosphorous is generally derived from the weathering of apatite^104^ and is then converted to available phosphate by plants and microorganisms^105^. Phosphorous tends to adsorb onto other substances such as iron-containing minerals within the soil and is, therefore, often found in low concentrations with varying biological availability^106^. Phosphorous has been identified as one of the drivers of microbial growth in Antarctica in studies on the South Shetland Islands^107^ and in Taylor Valley in Victoria Land^108^. It is also a key limiting nutrient in Antarctic fellfield environments, such as on Signy Island^109^ and the South Shetland Islands^110^. Phosphorous is similarly a limiting nutrient in the Arctic^106^, including for plant growth^111^. Darcy et al. (2018) highlighted that phosphorous, rather than nitrogen, can be the key limiting nutrient to environmental succession after glacier retreat^112^. Given that the death and decomposition of arthropods can increase soil phosphorous levels^113^, any increase in numbers or biomass of non-native (or native) invertebrates under climate change could increase availability of this nutrient. Furthermore, increased human activity in the polar regions may result in increased organic matter and higher phosphorous levels^114^ which may, in turn, reduce nutrient barriers to the establishment of future non-native species. To date, however, phosphorous has not been quantified in any field-based studied assessing the impacts of non-native species in polar regions (and only in one laboratory-based study^115^). We suggest that this nutrient be prioritised in future research, as it is vital for plant growth and function^116^, as well as affecting microbial communities.

### Impacts of non-native invertebrates

Within an indoor mesocosm experiment in the Arctic (northern Sweden), Blume-Werry et al. (2020) identified that non-native earthworms (*Lumbricus* sp. and *Aporrectodea* sp.) increased nitrogen mineralisation in the soil, resulting in a 50% increase in nitrogen concentration in the grass species, *Festuca ovina*^97^. These worms fragment dead vegetation making it available to soil microbial communities that can then mineralize it, in turn releasing nutrients that become available for microbial and plant uptake^117^. Blume-Werry et al. (2020) also reported that, although there was an increase in nitrogen mineralisation, nitrate, ammonia and phosphorous concentrations did not differ significantly between earthworm-influenced and control sites. In addition, the abundance of native plant species did not change during the course of their study, perhaps due to the static nutrient availability. However, the height of the floral shoots in another grass species, *Deschampsia flexuosa*, increased (by up to a factor of 3 in a meadow environment) and the number of floral shoots in *F. ovina* more than doubled in the earthworm treatments. In a parallel water leaching experiment, the authors noted that earthworms increased the ammonia concentration by over four orders of magnitude.

Another example of a non-native invertebrate affecting biologically available nitrogen comes from the maritime Antarctic. The chironomid midge, *Eretmoptera murphyi* (Fig. 2d), was accidentally introduced in the 1960s to a site adjacent to Signy Island research station (Fig. 2e) from its native habitat in sub-Antarctic South Georgia where the species is palaeoendemic^101,118^. In the last three decades, *E. murphyi* has expanded its range significantly on the island^101^, and its detritivorous larvae can achieve rates of moss peat breakdown several times greater than that of the entire native microarthropod community^119^. Bartlett et al. (2023) found that *E. murphyi* larvae were associated with a significant 3–5× increase in biologically-available soil inorganic nitrogen levels, with 5.22 mg/L inorganic nitrogen recorded in *E. murphyi* sites versus 0.67 mg/L in control sites. Nitrate levels were also significantly increased where *E. murphyi* larvae were established, 4.22 mg/L versus 0.26 mg/L at sites where larvae were absent. These results indicate that *E. murphyi* larvae increased nitrogen availability to levels similar to those recorded around vertebrate wildlife-exposed sites on Signy Island. The study reported a significant positive correlation between *E. murphyi* abundance (individuals per square metre) and lichen percentage cover, and a negative correlation with non-*Polytrichum* moss species cover. However, there was no significant correlation between *E. murphyi* abundance and *Polytrichum* moss species, or with the abundance of any native invertebrates (Collembola and Acari). It is possible that other factors not measured in this study, e.g. phosphorous availability, could limit changes in plant growth. Also, it could take more than 60 years in slow-functioning polar systems to see significant community changes. Nonetheless, these altered nutrient conditions seem likely to impact plant growth and may provide pathways facilitating future non-native species establishment.

As previously noted, over 200 non-native species are confirmed to be established on sub-Antarctic islands^6,25^, and some of these now represent potential introduction threats to sites further south^25,120^. On sub-Antarctic Marion Island (Fig. 2f), the non-native midge, *Limnophyes minimus* (Fig. 2g), has significantly increased litter turnover. with larvae achieving rates of up to 8.54 g m^−^^2^ y^−^^1^ (dry mass ingestion rates) compared to the maximum of 6.86 g m^−^^2^ y^−^^1^ achieved by larvae of the native flightless moth, *Pringleophaga marioni*^121^. The woodlouse *Porcellio scaber* (Isopoda) has also been introduced to Marion Island^122^ and its large size suggests it could have significant impacts on nutrient cycling. To test this hypothesis, Martin et al. (2023) conducted a laboratory-based experiment where they introduced *P. scaber* to soil mesocosms containing the native Antarctic collembolan, *Cryptopygus antarcticus*, to see if it would affect nutrient availability, seed germination and growth of the non-native grass *Poa pratensis*^115^. In addition, they investigated the impact of a euedaphic non-native springtail, *Folsomia candida*, which has been reported from small areas of geothermally active ground and associated vegetation on Deception Island^35,123^. The study examined five different combinations of invertebrate communities with increasing complexity under current mean maritime Antarctic soil surface conditions (2 °C) and a future warming scenario (7°C). Warming to 7 °C strongly amplified biogeochemical responses, including significantly lower soil pH (mean = 5.8 vs. 6.0 at 2 °C) and substantial reductions in leachable nutrients (49% PO₄, 42% NO₃ + NO₂, 94% NH₄). The lowest pH values (5.5 ± 0.15) occurred when *P. scaber* and *C. antarcticus* were present together, indicating that invertebrate activity and warming jointly increased soil acidification. In contrast, no significant pH differences were detected at 2 °C, suggesting that warming amplified the biogeochemical influence of invertebrate activity. The authors also reported a near-doubling of total CO₂ fluxes under warming (from 7.7 to 14.7 µg CO₂ g⁻¹ soil), with *P. scaber* accounting for roughly half of this increase, implying that warming enhanced microbial and faunal respiration. It was concluded that the non-native woodlouse may facilitate the decomposition activity of the native springtail, although a longer study period (more than 83 days) would be required to confirm that it is not competing with *C. antarcticus*. When only the non-native *P. scaber* was present, *P. pratensis* nitrogen content increased from 2.35 ± 0.15% (in control mesocosm with no invertebrates) to 3.09 ± 0.23%. A smaller increase was seen when only the native *C. antarcticus* was present (2.15 ± 0.24% vs. 2.80 ± 0.16%). Although there was an increase in the uptake of nitrogen in *P. pratensis* when the invertebrates were present, this did not translate into increased plant biomass or growth - a similar result to that reported by Blume-Werry et al. (2020)^97^. This may be due to the particularly cold climate in Antarctica resulting in slow plant growth^124^, meaning longer periods are required to assess any growth effects.

The higher rates of decomposition achieved by *P. scaber* may be attributed to its larger body size compared to the other two species, leading to a greater energy requirement, so more organic matter is broken down and egested. Although not quantified in the literature, it is clear that a number of well-established non-native invertebrates in both the sub- and maritime Antarctic regions are larger than most or all of the native invertebrate fauna, with examples including *E. murphyi* on Signy Island^125^ (Fig. 2d, e), *Trichocera maculipennis* (Diptera: Trichoceridae) on King George Island^126^, *T. regelationis* (Diptera: Trichoceridae) on South Georgia^126^, invasive carabid beetles on South Georgia and Kerguelen^127^, non-native woodlice (*P. scaber*) on Gough and Marion Islands^128^ and terrestrial flatworms (*Arthurdendyus vergranids* and *Kontikia andersoni*: Tricladida Geoplanidae) on Macquarie Island^120,129,130^. Although there are only a few of these large species, they have the ability to make a large contribution to bioturbation. This highlights that such species should be a particular focus of research effort in future monitoring programmes. Importantly, maritime Antarctic islands do not host any native macro-invertebrates, unlike the sub-Antarctic islands, so the impact of invertebrate detritivore introductions on nutrient cycling and the wider terrestrial ecosystem seems likely to be more pronounced. However, larger species do not dominate lists of non-native invertebrates in polar regions. For example, Gaston et al. (2001) found that many non-native species were smaller than their native counterparts (although not consistently across groups)^131^. In a very recent study, (Hughes et al., 2025), it is apparent that Collembola and small Arachnida dominate the non-native invertebrates present in the maritime Antarctic^8^.

### Impacts of non-native plants

The non-native grass, *Poa annua* (Fig. 2h), is widespread in the sub-Antarctic and has also been introduced to the maritime Antarctic (where the largest population is present near the Polish Arctowski Station in Admiralty Bay on King George Island^6,125,132,133^. Laboratory and field experimental studies have suggested that this may have implications for native plant communities, such as decreasing the growth of the two native Antarctic vascular plants, *Deschampsia antarctica* and *Colobanthus quitensis*^133–135^. This is suggested to be because *P. annua* displays a higher level of phenotypic plasticity and also achieves a competitive advantage over the other species by altering the availability of nitrogen, by changing the community of root endophytes^136^. In contrast, Cavieres et al. (2018) reported that, under existing conditions of temperature and nitrogen availability in the maritime Antarctic, *P. annua* may be able to coexist with the native plant species while, with lower temperatures and nitrogen availability, *D. antarctica* was able to outcompete *P. annua*^137^. This result may be due to *P. annua*’s ability to take up nitrogen more rapidly than its competitors, local differences in the availability of soil nutrients (e.g. through the influence of local vertebrate concentrations), and other site-specific topographical features.

To date, *P. annua* is the only non-native plant species that has established in the maritime Antarctic and its success has been attributed to the presence of fungal endophytes that may have been introduced at the same time as the plant^138,139^. Molecular analyses identified the dominant fungal genus within *P. annua* tissues to be *Cladosporium*, which is well known for conferring enhanced tolerance to environmental stress through a range of physiological and molecular mechanisms. These include the synthesis of proline, a stress-related amino acid that mitigates oxidative damage by suppressing reactive oxygen species. Also, the upregulation of Late Embryogenesis Abundant (LEA) proteins, which stabilise membranes and proteins under desiccation and freezing conditions. Overall, these changes can increase environmental tolerance and, therefore, the rate of plant growth and seed germination, as well as enhance seed biomass^138,140–142^. Although *Cladosporium* was the dominant (95%) component of microbial diversity reported by Ballesteros et al. (2022), other rarer taxa were also present in *P. annua* and these fungal endophytes require further study to better understand their possible roles in the success of their host species. Additionally, the specific structures of the phytochemicals and secondary metabolites produced, and their biological effects, remain unclear^138^.

We located no published studies investigating the impacts of non-native plants and their associated microbiomes on soil nutrients in the High Arctic. An additional challenge in this regard is that many of the introduced species have arrived in combination with imported nutrient-rich soil^27^, meaning that it is impossible to disentangle species-specific effects. While relatively few plant species have established in the high-Arctic^143^, across the Arctic as a whole Wasowicz et al. (2020) reported 341 non-native vascular plants, of which 188 are considered persistent and 11 invasive^22^. Dominant genera include *Rumex*, *Poa*, *Ranunculus*, *Trifolium* and *Vicia*. The capacity of *Poa* spp. and their associated microbes to alter polar terrestrial communities and soil systems is considered above. Non-polar studies with invasive *Rumex* spp. have shown an ability to inhibit the growth of less competitive native species and to dominate soil cover, hence reducing overall plant diversity^144^. *Rumex* spp. can inhibit the growth of other plants through root exudates^145^. *Ranunculus* spp. can also impact native plants through competition and allelopathy, however, Masters and Emery (2016) found the primary mechanism of impact on native species was through competition for space and light^146^. Thus, a primary impact of *Rumex* or *Ranunculus* on soil biochemistry seems to be depleting nutrient resources at a rate more rapid than native species. In contrast, both the genera *Trifolium* and *Vicia* are legumes, with root microbiomes capable of fixing nitrogen. As set out above, while this can benefit native species it can also promote the establishment of other non-native plants. notably, some *Trifolium* spp. have also been shown to increase arbuscular mycorrhiza (AM) colonization, which then increases phosphorus uptake and growth in AM host plants^147^. *Vicia* spp. can also promote the proliferation and activity of beneficial soil bacteria that are involved in P cycling and can convert insoluble P into forms that plants can use^148^.

Finally, evidence is available that non-native plants can indirectly influence soil chemistry by altering soil faunal communities. For example, the grass, *Agrostis stolonifera*, on Marion Island (sub-Antarctic) is associated with a 50% reduction in the mean native plant communities (species richness) and an increase in macroinvertebrates^149^. On South Georgia (sub-Antarctic), the poorer nutritional quality of *P. annua* (low water and high nitrogen content) compared to native grasses impaired growth in the native beetle *Hydromedion sparsutum*, resulting in a decline in abundance^150^. On the Kerguelen Islands (sub-Antarctic), Badenhausser et al. (2022) reported generally higher abundances of native macroarthropods, but a reduction in the number of native microarthropods in patches dominated by a variety of non-native vegetation, such as the bluegrass *P. pratensis*, suggesting plant-driven changes in litter quantity or alterations to abiotic conditions may underlie these effects^151^. These shifts in faunal community composition could feed back into soil nutrient cycling (e.g. via altered decomposition, nutrient mineralisation or bioturbation), providing an additional pathway through which non-native plants may affect soil chemistry in polar regions.

## The need for standardised approaches

One challenge to comparing studies on the impacts of non-native species on soil properties is the use of different methodologies. This does not apply to polar studies alone and is an important consideration for such investigations globally. These differences make long-term monitoring of the impacts of existing invasions on ecosystem functioning less robust and insightful. In the studies considered here, key methodological differences include the ways in which soil nutrients are extracted - such as ion exchange membranes to quantify available nutrients^101^, and water extraction to observe leachable nutrients^115^. Furthermore, the nutrients analysis methodologies also vary, for instance continuous flow analysis^101^, isotope ratio mass spectrometry and elemental analysis^97^ and autoanalysis^115^. Although these differences reflect variations in study aims and equipment availability, the need for approaches that provide directly comparable data is clear. Some research has demonstrated how differences in extraction reagents and specific protocol details, such as shaking times and the ratio of soil mass to extraction reagent volume, can impact quantification of soil nutrients^152^. Therefore, a clear understanding of the underlying chemistry is essential in selecting methodologies that are appropriate for the soil type considered and comparable across studies^153^. In particular, peat soils containing high levels of organic carbon are common in some polar regions^154,155^ and, due to the high water content of these soils, consideration of the volume of extraction reagent and dilution factor before analysis is required^156^. This is perhaps even more relevant in the polar regions where nutrients are often (very) limited and the use of extraction methods that provide an accurate nutrient yield are particularly important. In addition to standardised methodological approaches, improved protocols are needed to detect and document non-native species during the early stages of colonisation. As recommended by Hughes & Convey (2012), more comprehensive baseline data on Antarctic biodiversity (also applicable to the Arctic) are required to identify newly established non-native species and to monitor existing ones^157^. Focused monitoring at high-risk locations - such as scientific stations and visitor sites, alongside improved assessment of propagule transfer routes will strengthen both prevention and early detection. Overall, more coordinated, rapid and standardised biosecurity protocols are needed across the polar regions to protect these fragile terrestrial ecosystems.

## Conclusions

**Fig. 3:**
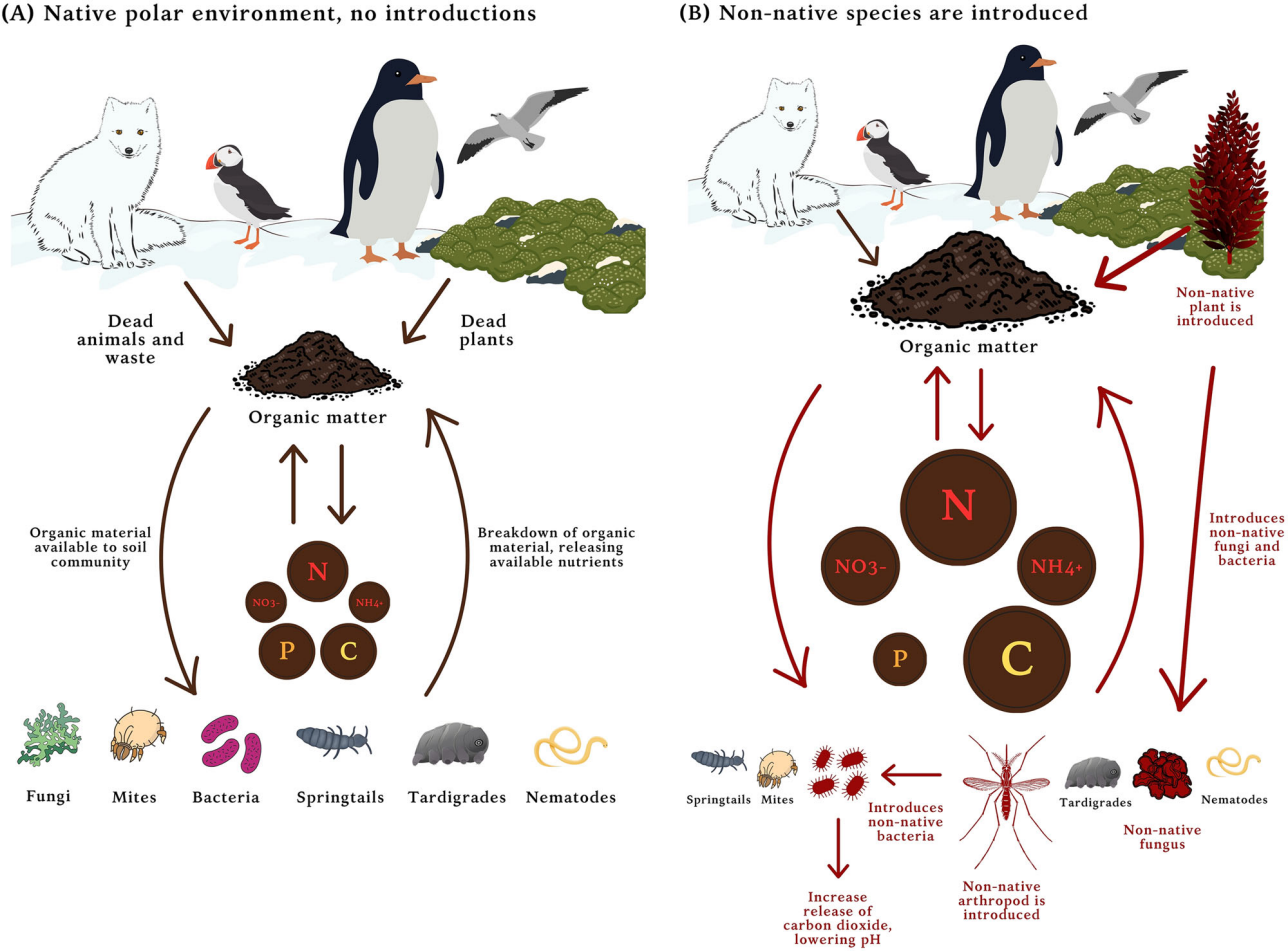
Conceptual illustration of potential impacts of introducing non-native arthropods and plants on the properties of polar soils, particularly in the context of increasing the amount of soil nutrients. Organisms illustrated in red are non-native species. Red arrows indicate the processes and interactions that are affected by non-native species. The size of the nutrients in each panel indicates increases under the influence of non-native species. There is currently no evidence of change to phosphorous levels. **A** Organic material enters the soil from dead plants, marine and terrestrial vertebrates, for example, Arctic foxes and puffins in the Arctic and penguins and seabirds in the Antarctic, and from their associated waste products, such as urine and guano. This supports the growth of soil microbes and other decomposers which break down the organic matter, releasing available nutrients into the soil, including nitrogen (N), nitrate (NO_3_^-^) ammonium (NH_4_^+^), phosphorous (P) and carbon (C). **B** Introduction of a non-native plant increases the quantities of organic material and, consequently, nutrients available to soil communities, resulting in increased microbial biomass and decomposition. This new plant species may also be associated with non-native fungi and elements of the microbiome, giving the plant a competitive advantage in its new habitat. Similarly, a non-native arthropod introduction can accelerate decomposition and nutrient turnover, altering soil processes and microbiome composition.

The introduction of non-native species to the polar regions represents a growing ecological challenge. Figure 3 conceptually illustrates potential impacts within polar soil systems. Although only a small proportion of arrivals are likely to establish in such nutrient-limited and extreme environments, evidence from several such successful events shows measurable impacts on soil chemistry and functioning, including increases in nitrogen, ammonia, nitrate and carbon concentrations. These changes indicate that non-native plants and invertebrates are already influencing key biogeochemical cycles. Key to understanding these impacts is the requirement to examine the microbiome associated with non-native introductions. This is a neglected aspect of invasion biology generally and is especially important in the polar regions, where there is a unique microbial diversity that is currently poorly characterised and under-represented in the literature^54^; we need to know what is there already to understand what is being introduced. Much more detailed soil microclimate data are required, with high spatial and temporal resolution, to more effectively model the capacity of non-native species (including microorganisms) to establish, spread and change terrestrial ecosystems. In addition, further research is required to develop in-depth understanding of invasion mechanisms, emphasising the need for stricter biosecurity protocols that take microbial communities into consideration.

Most non-native species currently present in the Arctic are concentrated around human settlement sites, whereas the distribution of some in the Antarctic has expanded beyond the immediate vicinity of these sites and into natural habitats, particularly in the sub-Antarctic. Introduced species that have nitrogen-fixing, phosphorous cycling and/or decomposition abilities can make an important contribution in creating favourable conditions to further facilitate potential non-native species establishment. With ongoing warming and increasing human activity, the combination of climate and biological change poses an escalating threat to the uniquely adapted polar biota. Prioritising the protection of soil health through coordinated biosecurity measures, long-term monitoring and standardised biogeochemical assessments will be essential to detect and manage these impacts before they become irreversible.

## Data Availability

No datasets were generated or analysed during the current study.
